# T-cell lymphoma with rhabdomyolysis: case report and literature review

**DOI:** 10.1590/1414-431X20176278

**Published:** 2018-10-08

**Authors:** Zhao-Zhong Ouyang, Ting-Sheng Peng, Qing-Hua Cao, Yin Ouyang, Jun-Xun Li, Qiu-Xiang Wei, Hong Zhan

**Affiliations:** 1 Department of Emergency, The People Hospital of Jiangmen City, Jiangmen, China; 2 Depatment of Pathology, The First Affiliated Hospital, Sun-Yat-Sen University, Guangzhou, China; 3 Department of Laboratory Medicine, The First Affiliated Hospital, Sun-Yat-Sen University, Guangzhou, China; 4 Department of Emergency, The First Affiliated Hospital, Sun-Yat-Sen University, Guangzhou, China

**Keywords:** Rhabdomyolysis, Multiple organ failure, T-cell lymphoma, Hemophagocytic lymphohistiocytosis, Case report

## Abstract

Rhabdomyolysis refers to the destruction or disintegration of striated muscles. This syndrome is characterized by muscle breakdown and necrosis, resulting in the leakage of intracellular muscle constituents into the circulation and extracellular fluid. We report a rare case of rhabdomyolysis complicating multi-organ failure caused by T-cell lymphoma in a 32-year-old woman. The final diagnosis was rhabdomyolysis caused by peripheral T-cell lymphoma based on bone marrow aspirate and biopsy.

## Introduction

Rhabdomyolysis refers to the destruction or disintegration of striated muscles. This syndrome is characterized by muscle breakdown and necrosis, resulting in the leakage of intracellular muscle constituents into the circulation and extracellular fluid. Rhabdomyolysis ranges from an asymptomatic illness with elevated creatine kinase (CK) levels to a life-threatening condition associated with extreme elevated levels of CK, electrolyte imbalance, acute renal failure (ARF), and disseminated intravascular coagulation (1). The cause of rhabdomyolysis is usually easily identified. However, in some instances, its etiology is elusive. Muscular trauma is the most common cause of rhabdomyolysis. Less common causes include muscle enzyme deficiencies, electrolyte abnormalities, infectious causes, drugs, toxins, and endocrinopathies. Rhabdomyolysis is commonly associated with myoglobinuria. If this becomes sufficiently severe, it can result in ARF, weakness, myalgia, and tea-colored urine, which are the main clinical manifestations. The most sensitive laboratory finding of muscle injury is creatine phosphokinase, and a level greater than 5000 U/l indicates serious muscle injury in the absence of myocardial or brain infarction. The management of patients with rhabdomyolysis includes advanced life support (airway, breathing, and circulation), followed by measures to preserve renal function. The latter includes vigorous hydration. The use of alkalizing agents and osmotic diuretics, which are commonly used, remains with unproven benefits.

## Case Report

On March 7, 2013, a 32-year-old woman diagnosed with rhabdomyolysis-complicated ARF was admitted to the Department of Emergency, the First Affiliated Hospital, Sun Yat-sen University with complaints of fever, loss of appetite, general fatigue, and sudden muscle weakness. Fifteen days earlier, she presented to a local hospital with fever, chills, abdominal pain, nausea, vomiting, diarrhea, general fatigue, and sudden muscle weakness without other symptoms or signs. Examination revealed fever, acute kidney injury, hepatic lesion, coagulopathy, and severe anemia. After the preliminary assessment, it was found that rhabdomyolysis was caused by an infectious disease and complicated with multiple organ failure and with possible sepsis. She was rehydrated, transfused and covered with wide-spectrum antibiotics (meropenem), but these treatments did not show any marked improvement.

She was immediately transferred to our emergency department for further evaluation and treatment. Upon arrival, physical examination confirmed the presence of muscle weakness, with muscle strength grade of 2 to 3. Laboratory abnormalities were identified including markedly elevated CK levels that peaked at 8024 IU/L, a Cr level of 37.5 mg/dL, an elevated liver level of the enzyme alanine aminotransferase of 104 U/L, a mild elevated glutamic-oxaloacetic transaminase level of 39 U/L, as well as an activated partial thromboplastin time of 39.2 s, a decreased fibrinogen level of 0.67 g/L, and pancytopenia. Furthermore, chest X-ray examination revealed left lower pneumonia, while abdominal ultrasound examination revealed hepatosplenomegaly. In addition, ultrasound revealed enlargement of retroperitoneal lymph nodes.

As a result, ARF caused by rhabdomyolysis was diagnosed, and treatment was initiated with hydration, continuous hemodiafiltration, and urine alkalization, resulting in significant improvements in physical strength and renal function (Cr=19.5 mg/dL) and decreased CK levels that peaked at 136 IU/L. However, the cause of rhabdomyolysis remained unclear. Recurrent fever and hemophagocytic syndrome were noted. Serum ferritin level elevated dramatically to 40,000.00 ng/mL. Multiple laboratory studies were ordered. Bone marrow aspirate and biopsy were performed on hospital days 5 and 7 to rule out the infiltrative process. Methylprednisolone pulse therapy resulted in moderate improvement of the patient’s general condition.

**Figure 1:**
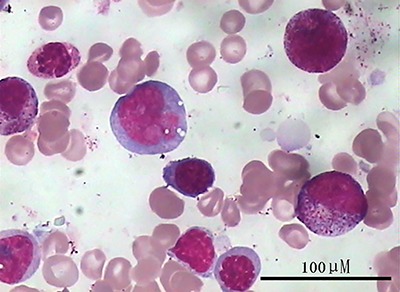
Hyperplasia of bone marrow, decreased granulocyte ratio, increased erythroid proportion, containing 12% unclassified cells. Bar: 100 μm.

On admission, the patient was covered with broad-spectrum antibiotics. Her renal and hematological system functions continued to deteriorate, which required hemodialysis and multiple transfusions. Pancytopenia worsened. She progressed to multi-organ failure and needed bi-level airway pressure ventilation. On hospital day 9, the patient was discharged due to treatment abandonment. Six hours after discharge from the hospital, she expired at home without a definitive premortem diagnosis. The results of the examinations of the bone marrow aspirate and biopsy are shown in Figures 1–3.

**Figure 2 f02:**
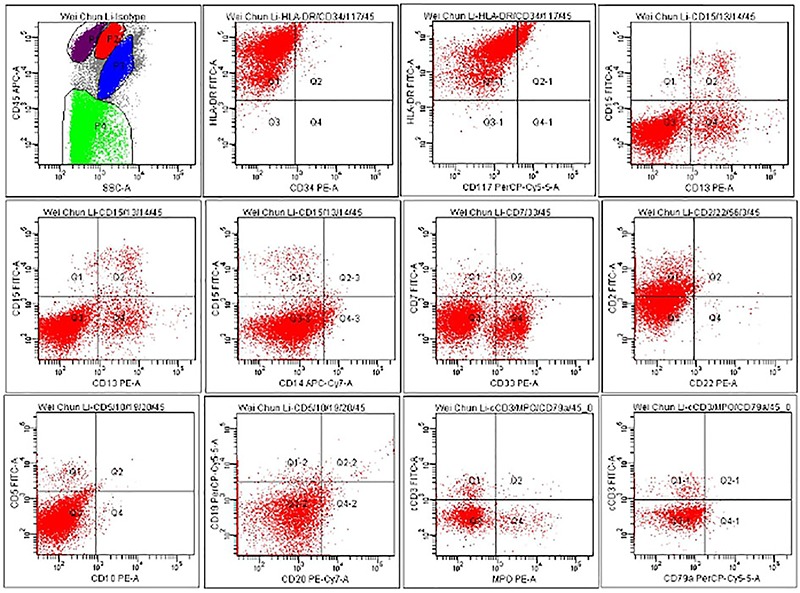
FCM-antigen results for HLA-DR=99.5%, CD13=13.5%, CD33=24.4%, CD2=37.8%.

**Figure 3:**
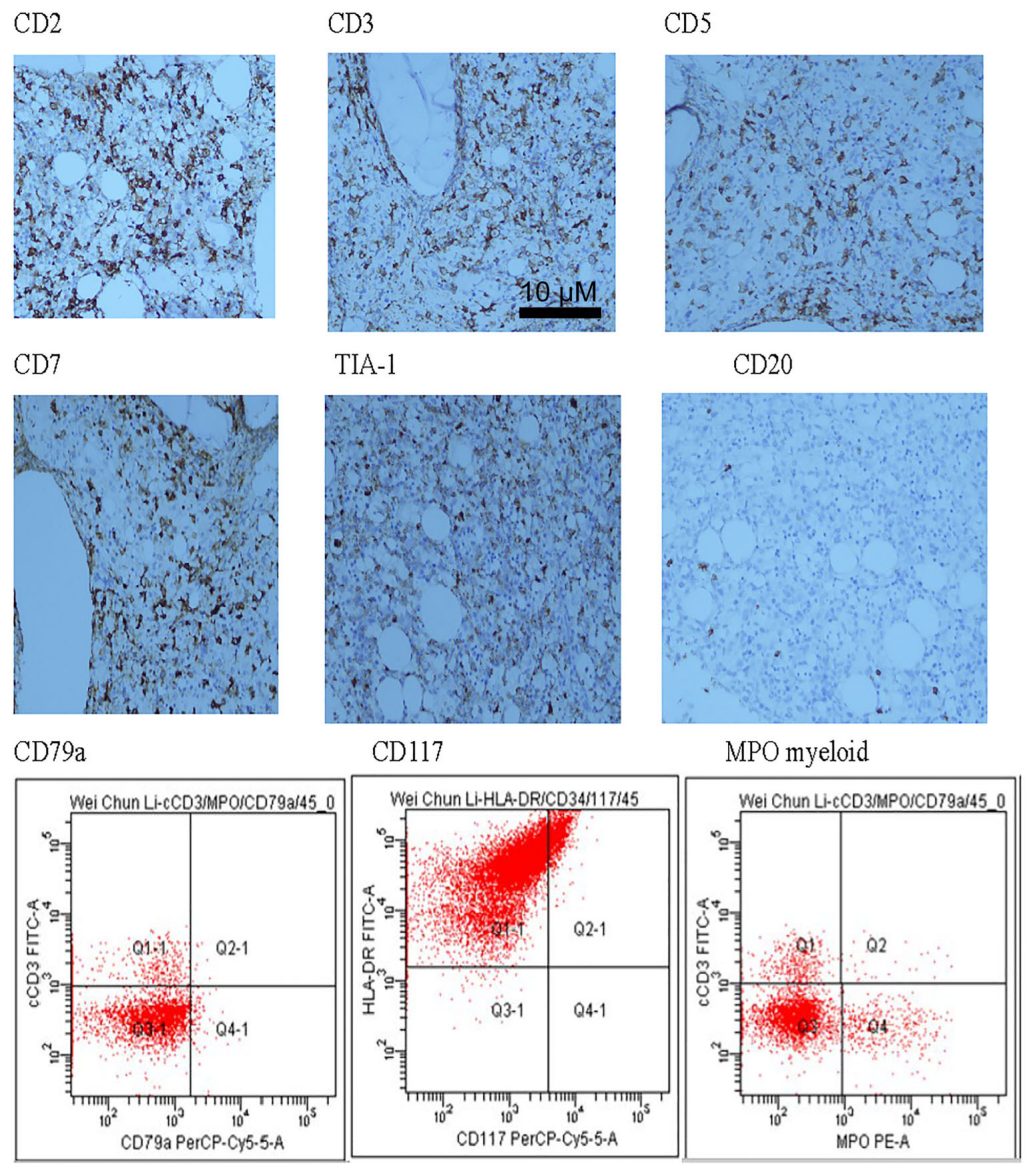
Pathologic and immunohistochemical analysis showing hematopoietic tissue between bone marrow scattered mass. Specific cells with patchy distribution, in which cells ranged in size from small to medium, nuclei were pale, enlarged, and irregular including thin karyotheca, inconspicuous nucleoli, partly visible nucleoli, and interstitial fibrous tissue hyperplasia. Atypical cells CD2, CD3, CD5, CD7, and TIA-1 were found positive, CD20, CD79a, and CD117 were found negative, and MPO myeloid cells were found positive. Bar: 10 μM. Diagnosis: Lesions conformed to abnormal proliferation of bone marrow T lymphocytes. The case was considered to be T-cell lymphoma involving the bone marrow.

### Pathology and immunohistochemistry

Hematopoietic tissues in the bone marrow contained a scattered mass of specific cells with a patchy distribution. These cells sized from small to medium, the nuclei were pale, enlarged and irregular, including a thin karyotheca, inconspicuous nucleoli, a partly visible nucleoli, and interstitial fibrous tissue hyperplasia.

### Immunohistochemistry

Atypical cells CD2, CD3, CD5, CD7, and TIA1 were found positive, CD20, CD79a, and CD117 were found negative, and MPO myeloid cells were positive.

### Diagnosis

Lesions conformed to the abnormal proliferation of bone marrow T lymphocytes. The diagnosis was considered to be T-cell lymphoma involving the bone marrow.

## Discussion

Rhabdomyolysis is a well-known clinical syndrome of muscle injury associated with myoglobinuria, electrolyte abnormalities, and often acute kidney injury (AKI) (2). Typical symptoms include pain, weakness, tenderness, and/or swelling of the injured muscles. It can also present with ambiguous symptoms of fatigue, nausea, vomiting, and fever.

T-cell lymphoma and natural-killer-(NK-) cell lymphoma represent the smaller subsets of non-Hodgkin’s lymphoma (NHL) that appear to have a geographical predilection for Asia. In Europe and North America, T-cell and NK-cell lymphoma account for 5–10% of all cases of NHL whilst in Asia this percentage is as high as 24% (3). T-cell lymphomas, as a group, carry a poorer prognosis compared to their B cell counterpart (4). In the subgroup of patients with a low international prognostic index (IPI) score of 1-2, 5-year overall survival was 55% in those with T-cell lymphomas and 71% in those with B-cell lymphomas, and this difference in survival was also reflected in patients with higher IPI scores (5). T-cell lymphomas, however, represent a heterogeneous group of diseases with variations in clinical characteristics, prognosis, and response to treatment.

NHL commonly presents as extranodal disease. Although the definition of primary extranodal lymphoma is somewhat controversial, the commonly accepted definition is involvement of an organ with no or minor local lymph node enlargement (6). Infiltration of the muscle is a very uncommon manifestation of lymphoma and most commonly occurs in the gluteal and pelvic musculature as a result of hematogenous dissemination or direct spread from adjacent lymph nodes or bone; primary involvement of the muscle is exceedingly rare (7). By 1997, fewer than 50 cases of primary muscle lymphoma had been described (8). Primary muscle lymphoma was found to account for just 0.1% of over 7000 cases of lymphoma diagnosed over a 10-year period at the Mayo Clinic (8). By comparison, in a study of 1168 patients with NHL, <1% involved the ovary and 3% involved bone (6)

In this report, we demonstrated a rare case of rhabdomyolysis, which caused T-cell lymphoma multi-organ failure. The potential for rhabdomyolysis to represent a sign of lymphoma should be considered, and attention must be given on the occurrence of lymphoma.
